# AMBRA1 and its role as a target for anticancer therapy

**DOI:** 10.3389/fonc.2022.946086

**Published:** 2022-09-27

**Authors:** Xiang Li, Yuan Lyu, Junqi Li, Xinjun Wang

**Affiliations:** ^1^ Department of Neurosurgery, The Fifth Affiliated Hospital of Zhengzhou University, Zhengzhou University, Zhengzhou, China; ^2^ Henan Joint International Laboratory of Glioma Metabolism and Microenvironment Research, Henan Provincial Department of Science and Technology, Zhengzhou, China; ^3^ Medical Research Center, The Third Affiliated Hospital of Zhengzhou University, Zhengzhou University, Zhengzhou, China; ^4^ Department of Neurosurgery, The Third Affiliated Hospital of Zhengzhou University, Zhengzhou, China

**Keywords:** AMBRA1, autophagy, mitophagy, tumorigenesis, targeted therapy

## Abstract

The activating molecule in Beclin1-regulated autophagy protein 1 (AMBRA1) is an intrinsically disordered protein that regulates the survival and death of cancer cells by modulating autophagy. Although the roles of autophagy in cancer are controversial and context-dependent, inhibition of autophagy under some circumstances can be a useful strategy for cancer therapy. As AMBRA1 is a pivotal autophagy-associated protein, targeting AMBRA1 similarly may be an underlying strategy for cancer therapy. Emerging evidence indicates that AMBRA1 can also inhibit cancer formation, maintenance, and progression by regulating c-MYC and cyclins, which are frequently deregulated in human cancer cells. Therefore, AMBRA1 is at the crossroad of autophagy, tumorigenesis, proliferation, and cell cycle. In this review, we focus on discussing the mechanisms of AMBRA1 in autophagy, mitophagy, and apoptosis, and particularly the roles of AMBRA1 in tumorigenesis and targeted therapy.

## Introduction

The global incidence and mortality of cancers have been dramatically increasing annually (1). In recent decades, cancer has been a leading cause of death and severely impacted life expectancy worldwide (2). At present, the strategies of cancer treatment mainly include surgical excision, chemotherapy, radiotherapy, and immunotherapy. However, the results of these treatments are still unsatisfactory. The molecular and cellular mechanisms of cancer have been explored in the last decades, but metastasis, chemoradiotherapy resistance, and recurrence are still the key obstacles to cancer treatment (3–6). Consequently, there is a dire need of figuring out the underlying mechanisms of cancers and find ways to cure them.

Autophagy is a cellular process that regulates the degradation of its cytoplasmic components *via* lysosomes. There are three major autophagy pathways, including macro-autophagy, micro-autophagy, and chaperone-mediated autophagy (CMA), which mainly differ in delivery methods and wrapped cargoes. Macro-autophagy wraps and degrades intracellular cargoes through autophagosomes with a bilayer membrane structure by fusing with lysosomes eventually (7, 8), while mitophagy is a selective macro-autophagy for mitochondria decomposition (9). Micro-autophagy, compared with macro-autophagy, directly engulfs the organelles *via* lysosomal deformation (10). CMA degrades the KEFRQ motif-containing proteins with the help of a chaperone heat shock protein of 70 kDa (HSP70) (11). For the macro-autophagy (generally accepted as the term “autophagy”) process, the formation of autophagosome is mainly divided into initiation, nucleation, elongation, and maturation. Although autophagy has a controversial effect on tumors in a context-dependent manner (12), autophagy disorder impacts the initiation and progression of cancer. Therefore, autophagy may be a promising target for cancer therapy.

The activating molecule in Beclin1-regulated autophagy protein 1 (AMBRA1), identified as an autophagy-associated protein initially, is a fundamental factor in the process of autophagosome formation (13). Furthermore, AMBRA1 is an intrinsically disordered protein that accounts for its great plasticity, which enables it to be a splendid scaffold protein connecting other intracellular processes related to autophagy (14–16). Through multifarious molecular interaction techniques, such as mass spectrum, genetic engineering technology, co-immunoprecipitation (co-IP), and yeast two-hybrid screening, numerous interaction partners of AMBRA1 have been demonstrated, as summarized in **Table 1**. Therefore, not surprisingly, AMBRA1 participates in diverse physiological and pathological processes, for instance, embryogenesis, neural development, tumorigenesis and proliferation, differentiation, and epithelial–mesenchymal transition (EMT) (13, 26, 33–36, 42–45).

**Table 1:** The interaction partners of AMBRA1 protein.

The interaction protein of AMBRA1	Binding sites on AMBRA1	Function	Reference
BECLIN1	aa 533–751	Favoring the BECLIN1–Vps34 functional interaction	(13)
DLC1	aa 1075–1077 and 1087–1089	Inhibiting AMBRA1 and BECLIN1–VPS34 complex translocation to ER	(17)
Mito-BCL-2	The N-terminal and C-terminal region of AMBRA1	Harnessing AMBRA1 at mitochondria and inhibiting autophagy	(18)
Parkin	The N-terminal region of Ambra1	Local activation of class III PI3K around depolarized mitochondria	(19)
Caspases	D482 site in AMBRA1	Cleavage at D482	(20)
Calpains	?	Complete decomposition	(20)
TRAF6	aa 618–623 and 681–686	Supporting ULK1 ubiquitylation by LYS-63-linked chains	(21)
ULK1	The N-terminal and C-terminal region of AMBRA1	Activating AMBRA1 by phosphorylation	(21)
DDB1-CULLIN4 complex	The second AMBRA1 WD40 domain	Limiting AMBRA1 protein abundance and promoting AMBRA1 degradation	(22)
ELONGIN B-CULLIN5 complex	The C-terminal region of AMBRA1	Promoting the accumulation of the mTOR inhibitor DEPTOR	(22)
ELONGIN C-CULLIN5 complex	aa 735–1208	Negatively regulating the assembly and ubiquitin E3 ligase activity of CRL5 complexes	(23, 24)
RNF2	?	Ubiquitinating AMBRA1 at lysine 45	(25)
Catalytic subunit of PP2A	aa 275-281 and 1206-1212	Facilitating the dephosphorylation and degradation of the proto-oncogene c-Myc	(26, 27)
LC3	aa 1012-1023	Promoting mitophagy	(28)
FAK/Src	?	Regulating adhesion and invasive migration	(29, 30)
IKKα	Upstream of the LIR motif of AMBRA1	Promoting mitophagy	(31)
HUWE1	?	Promoting PINK1/Parkin-independent mitophagy	(31, 32)
ALDH1B1	?	Inhibiting carcinogenesis	(33)
Cyclin D	?	Regulating cell cycle	(34–36)
ATAD3A	?	Promoting PINK1 stability	(37)
Smad4	?	Facilitating TGFβ-driven metastasis	(38)
Cardiolipin	?	Promoting autophagosome formation	(39)
ERLIN1	aa 533-751 and 767-1269	Driving autophagy initiation	(40)
SUGT1	the C-terminal region of AMBRA1	Inhibiting the activity of CRL7 complexes	(22)
mTORC1	?	Inhibiting the activity of AMBRA1	(21)
CANX (calnexin)/GD3	?	Promoting autophagy	(41)
WIPI1	?	Promoting autophagy formation	(39, 41)
WASH	?	Promoting AMBRA1 degradation by potentiating RNF2	(25)
Akap8/Cdk9	?	Histone modifications and altered chromatin accessibility; transcriptional regulation	(29)

AMBRA1 plays multi-functional roles in intracellular physiological and pathological processes. The aberrant expression and dysregulation of AMBRA1 positively and negatively control tumor formation and progression through diverse signal pathways, such as c-MYC, cyclin D, mTOR, PI3K, STAT3, and TGFβ (21, 23, 24, 26, 34, 38). Thus, AMBRA1 may be a potential target biomarker for future cancer therapeutics.

## AMBRA1-protein structure and subcellular location

AMBRA1 was firstly identified by Francesco Cecconi using a gene-trap expression and mutational analysis to seek genes expressed in the development of the nervous system in 2007 (13, 46). *AMBRA1* gene is located in chromosome 11p11.2 with 24 exons, and it encodes a protein with a linear sequence of 1,298 amino acids. The subcellular location of AMBRA1 is mainly in cytoplasmic structures, such as autophagosome, cytoskeleton, endoplasmic reticulum, and mitochondria, and it is also found to be localized in the nucleus (13, 17, 18, 28, 29). Interestingly, the subcellular localization of AMBRA1 is dynamic, which primarily depends on autophagy induction. In the absence of autophagy induction, AMBRA1 tends to partially locate at mitochondria and cytoskeleton, and AMBRA1 re-localizes to the endoplasmic reticulum to enable autophagosome nucleation upon autophagy induction (17, 18).

**Figure 1:**
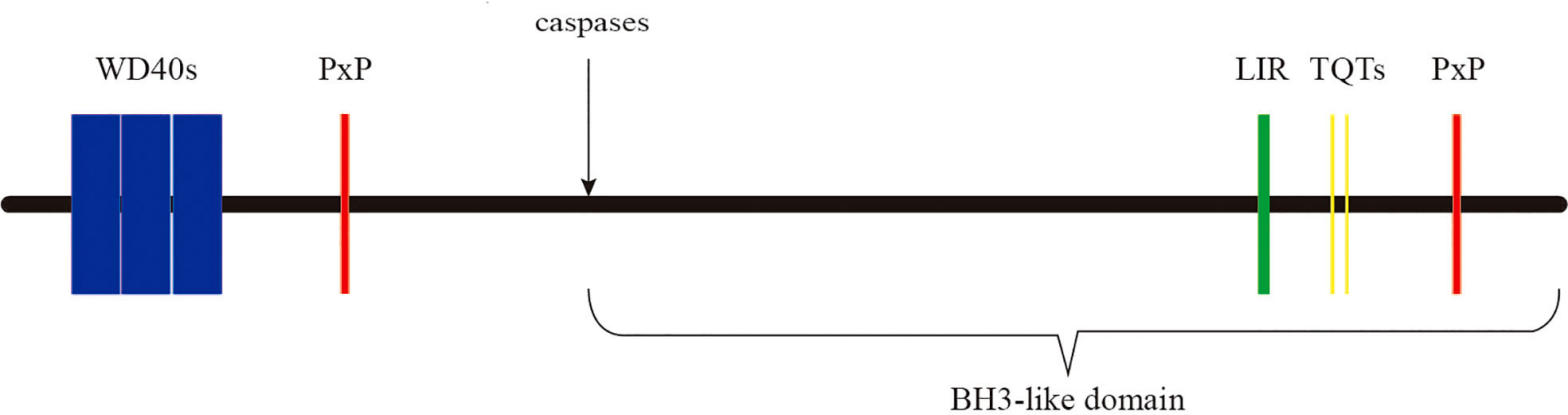
The domains and motifs of AMBRA1 protein. AMBRA1 contains WD40 domain (aa 1–175) and three kinds of motifs-two PxP motifs (aa 275–281 and 1206–1212), two TQT motifs (aa 1104–1106 and 1116–1118), and an LIR motif (aa 1043–1052). At the D482 site, AMBRA1 is cleaved by caspases.

AMBRA1 has no obvious domains but the WD40 domain at its N-terminus (13, 26). WD40 domain contains ~40 amino acids and acts as a binding site for the interaction of the protein with protein or DNA, so AMBRA1 can present a scaffold that assembles protein complexes or mediates transient interplay with other proteins (47). Furthermore, AMBRA1 contains 3 motifs-two PxP motifs, two TQT motifs, and one light chain 3 (LC3) interacting region (LIR) motif (**Figure 1**). The PxP motifs, corresponding to the aa 275-281 and aa 1177-1183 of AMBRA1, resemble the SH3 motif and bind with the catalytic subunit of protein phosphatase 2A (PP2A) to regulate c-MYC (26, 27). The TQT motifs located on the AMBRA1 C-terminal sequence mediate the interaction with the dynein light chain 1 (DLC1), fastening AMBRA1 to the dynein motor complex in the absence of autophagy induction (17). The LIR motif on its C-terminal region is critical for the binding between AMBRA1 and the autophagy-related protein 8 (ATG8) family proteins light chain 3 beta (LC3B) (28). Finally, AMBRA1 is cleaved by caspases at D^482^ during apoptosis (**Figure 1**). Its C-terminal part generates a BH3-like domain, called AMBRA1^CT^, which acts as a pro-apoptotic factor by directly binding and inhibiting anti-apoptotic factor B-cell lymphoma 2 (BCL2) (20, 48, 49).

## The role of AMBRA1 in autophagy initiation and apoptosis

In 1957, autophagy was first noted by Clark in the kidneys of neonatal mice by using an electron microscope (50) and firstly described by Deter and De Duve in the late 1960s without unveiling underlying mechanisms (51). In 1996, Oshumi and co-workers found ~30 autophagy-related genes (ATGs) in yeast (52, 53), which opened a new horizon for surveying this basic cellular process. Autophagy is a self-digestion process that engulfs impaired organelles or proteins to decompose into small molecules for cell reutilization, and this process is fundamental for cell survival.

**Figure 2:**
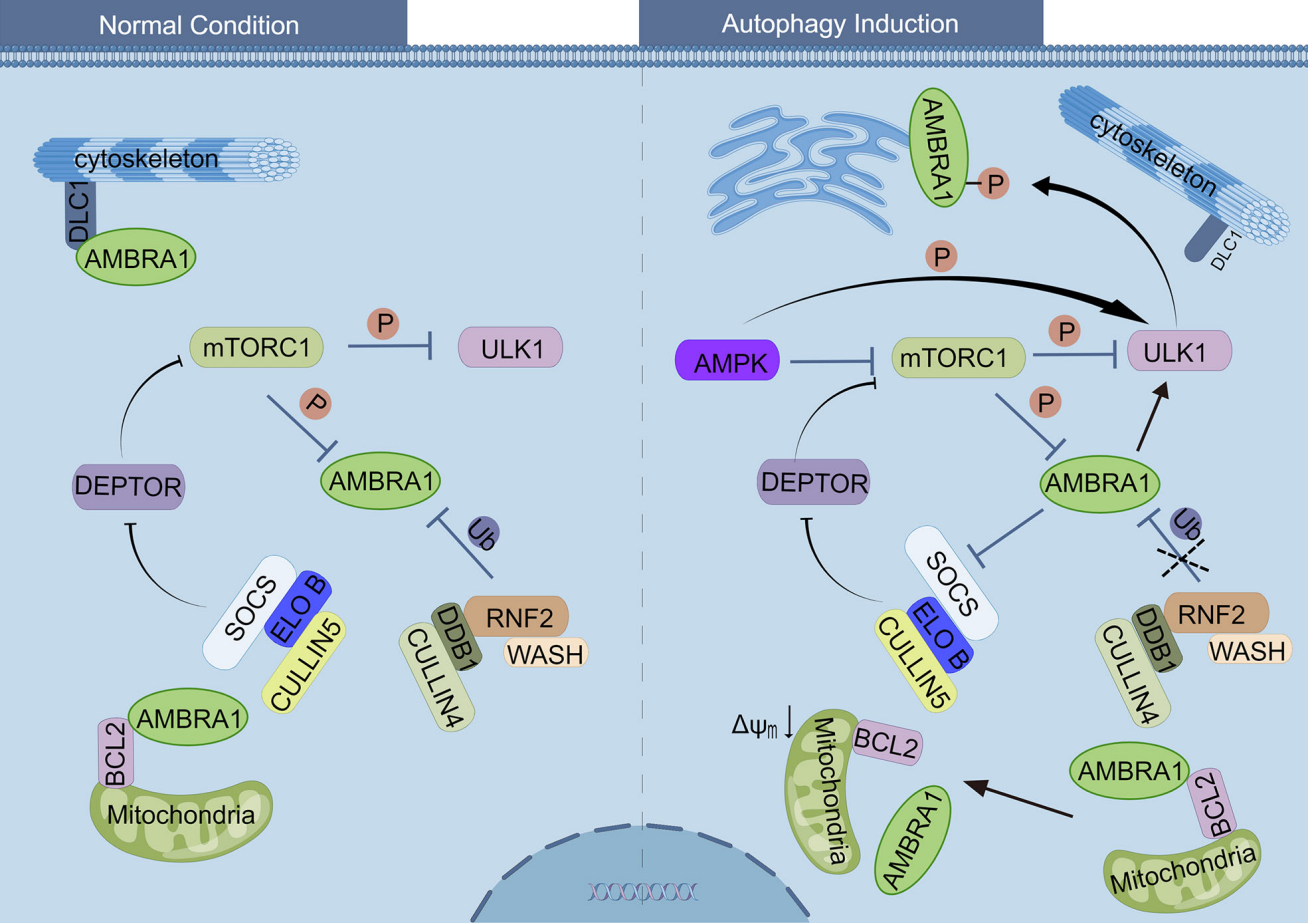
Regulation of AMBRA1 under normal conditions and autophagy induction. Left side: under normal conditions, AMBRA1 is relocated to the cytoskeleton through an interaction with DLC1, as well as with BCL-2 at the outer mitochondrial membrane. The mTORC1 inhibits ULK1 and AMBRA1 by phosphorylating ULK1 and AMBRA1, respectively. RNF2/DDB1/CULLIN4, together with WASH protein, ubiquitylates and degrades AMBRA1. SOCS/ELONGIN B/CULLIN5 ubiquitylates and degrades DEPTOR, resulting in activation of mTORC1 complex. Right side: upon autophagy induction of glucose starvation, AMPK inhibits mTORC1 and then reduces the phosphorylation of ULK1 and AMBRA1. Subsequently, AMPK directly interacts and activates the ULK1 by phosphorylation, and the dephosphorylated AMBRA1 ubiquitylates ULK1 to further promote the activity of ULK1. The activated ULK1 kinase phosphorylates AMBRA1, promotes its release from dynein motor complex, and relocates to mitochondria-associated membranes (MAMs). The degradation of AMBRA1 by RNF2/DDB1/CULLIN4 is inhibited, and therefore, AMBRA1 promotes DEPTOR accumulation and inhibits mTORC1 activity. The interaction between AMBRA1 and mito-BCL-2 is disrupted when mitophagy induction.

The paralleled levels of AMBRA1 and autophagy suggest that AMBRA1 is one of the pivotal proteins regulating autophagy. Under normal conditions, AMBRA1 remains in a low or dormant state: 1) AMBRA1 preferentially binds to BCL2 at the outer mitochondrial membrane (18); 2) AMBRA1 is a vital component of the BECLIN1/VPS34 complex, which is harnessed to the cytoskeleton through an interaction between the AMBRA1 and DLC1 (13, 17); 3) mTORC1 phosphorylates and inhibits AMBRA1 and ULK1, a protein kinase responsible for the recruitment of ATG proteins to the pre-autophagosomal structure. Furthermore, the DEP Domain Containing MTOR Interacting Protein (DEPTOR), an inhibitor of mTOR activity, is degraded by SOCS/ELONGIN B (ELO B)/CULLIN 5 (21, 22, 25, 54) (**Figure 2**). All the above processes prevent the activation phosphorylation of AMBRA1. Upon autophagy induction by glucose starvation, AMP-activated protein kinase (AMPK) inhibits mTORC1 through the phosphorylation of Tuberous Sclerosis 2 (TSC2) and Raptor with the result of reducing phosphorylation of ULK1 on Ser 757 and phosphorylation AMBRA1 on Ser 52 (21, 54). The phosphorylation of ULK1 on Ser 757 is reduced, and subsequently, AMPK directly interacts with and activates the dephosphorylated ULK1 by phosphorylating ULK1 on Ser 317 and Ser 777 (54). Moreover, the dephosphorylated AMBRA1/TRAF6 ubiquitylates ULK1 on Lys 63 to further promote ULK1 self-association, stability, and activity (21). The activated ULK1 kinase phosphorylates AMBRA1 and promotes its release from the dynein motor complex and relocates to mitochondria-associated membranes (MAMs) of the endoplasmic reticulum by interacting with CANX, GD3, WIPI1, ERLIN1, and Cardiolipin to enable autophagosome formation (17, 39–41, 55) (**Figure 2**). The exact phosphorylation site of AMBRA1 by ULK1 is unknown. As for the release of AMBRA1 from mitochondria upon autophagy induction, the underlying mechanism remains elusive, although ULK1 might be involved. In the autophagy induction stage, AMBRA1 not only regulates the activity of ULK1 kinase but also interacts with BECLIN1 and VPS34 and modulates their activity. In 2007, Gian Maria Fimia and colleagues firstly observed that AMBRA1 directly interacts with BECLIN1 and VPS34, and the downregulation of AMBRA1 markedly reduces BECLIN1-associated autophagy because of the reduced interaction between BECLIN1 and VPS34 (13). This corresponds to the characteristics of AMBRA1 as a scaffold protein that offers a platform for BECLIN1 and VPS34 interaction. To further identify the biological functions of AMBRA1, Antonioli et al. performed tandem affinity purification (TAP), sodium dodecyl sulfate–polyacrylamide gel electrophoresis (SDS-PAGE), and mass spectrometry to identify the interacting proteins of AMBRA1, and they found that some Cullin-RING ligase (CRL) components such as Cullin4, Cullin5, DNA damage-binding protein 1 (DDB1), Elongin B, and suppressor of G2 allele of SKP1 homolog (SUGT1) interact with AMBRA1, indicating that AMBRA1 is involved in mediating CRL ubiquitination activity (22). The temporal dynamic interaction of AMBRA1 with CULLIN 4 and CULLIN 5 regulates both the initiation and termination stages of autophagy, which keeps the autophagy under control.

**Figure 3:**
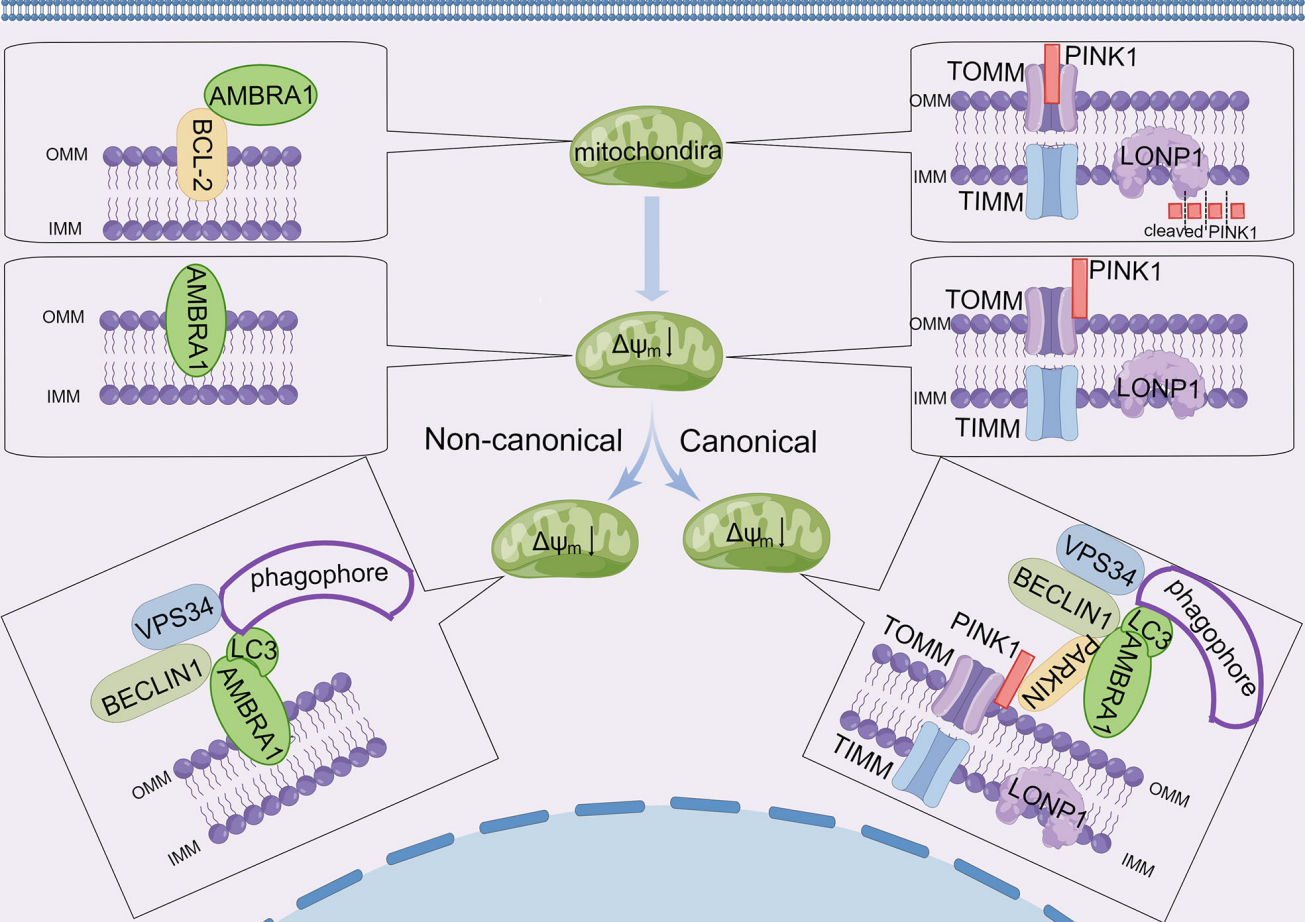
AMBRA1 and canonical/non-canonical mitophagy. Under normal conditions, AMBRA1 interacts with BCL-2 at the outer mitochondrial membrane. PINK1 is transported to the inner mitochondrial membrane through the TOM/TIM complex, and then PINK1 is damaged by LONP1. Upon loss of mitochondrial membrane potential (Δψm), AMBRA1 separates with BCL-2 and relocates to OMM. PINK1 accumulates on the outer membrane surface where it associates with the TOM complex. AMBRA1 promotes mitophagy of damaged mitochondria in two major ways: i) in non-canonical mitochondrial clearance, AMBRA1 functions as a mitophagy receptor and accumulates on the OMM, promoting specific binding to LC3 through a conserved LC3-interacting region (LIRs) and regulating the formation of phagophore enclosing mitochondria. ii) In canonical mitochondrial clearance, the accumulation of PINK1 recruits cytosolic PARKIN and AMBRA1, which induces new phagophores through its effect on VPS34 and its LIRs.

Selective engulfment of impaired mitochondria *via* autophagy, namely, mitophagy, is important for the efficient turnover of mitochondria. Experts found that the processes of mitophagy are mainly divided into receptor-mediated mitophagy and ubiquitin-mediated mitophagy in mammals. Emerging evidence indicates that AMBRA1 plays an important role in both processes (28, 56). As mentioned above, AMBRA1 preferentially interacts with mitochondrial BCL-2 (mito-BCL-2) in normal conditions, and the interaction between AMBRA1 and mito-BCL-2 is disrupted when mitophagy is activated (18). Van Humbeeck et al. revealed that AMBRA1 is a non-substrate interactor of E3 ubiquitin ligase Parkin, and the interaction of AMBRA1 and Parkin is enhanced upon mitochondrial depolarization, leading to the clearance of mitochondria in a Parkin-mediated manner (19). By analyzing the protein sequence of AMBRA1 and validating by immunoprecipitation study and point mutation of AMBRA1, Strappazzon and colleagues disclosed that AMBRA1 contains a LIR motif responsible for binding with LC3 in its C-terminus (28) . They also originally constructed a plasmid encoding myc-AMBRA1 fused to Actin assembly-inducing protein (ActA) that can target the AMBRA1–ActA protein to the outer mitochondrial membrane, ultimately proving that AMBRA1 acts as a powerful mitophagy regulator through Parkin-mediated and Parkin-independent mitophagy (28) (**Figure 3**). For Parkin-mediated mitochondrial clearance, the loss of mitochondrial membrane potential rapidly recruits AMBRA1 to the outer mitochondrial membrane (OMM), where it interacts with ATAD3A–TOMM–PINK1 complex to prevent PINK1 degradation by mitochondrial matrix protease Lon Peptidase 1 (LONP1). Then the increase of PINK1 on the OMM recruits Parkin from the cytosol to damaged mitochondria, leading to mitochondria clearance in a Parkin-mediated manner (37). In terms of PARKIN-independent mitophagy, AMBRA1 acts as a mitochondrial receptor, and E3 ubiquitin ligase HUWE1 promotes the LIR motif of AMBRA1 unfold to interact with LC3 (28, 31). However, for the origin of AMBRA1 in mitophagy, few studies have been conducted. It is speculated that mitophagy regulation by AMBRA1 may be attributed to the dissociation of mito-BCL-2. A study from Strappazzon and colleagues found that GSK-3β phosphorylates MCL-1 to release AMBRA1, while HUWE1 promotes MCL-1 degradation in Hela cells and MCF7 breast cancer cells (32), highlighting the need for further studies to elucidate molecular mechanisms of AMBRA1 and mito-BCL-2 in mitophagy. In conclusion, AMBRA1 plays a pivotal role in regulating mitophagy.

The interplay between autophagy and apoptosis is complicated. The stimulus factors for autophagy and apoptosis are similar, but the diverse outcomes may be due to different sensitivity thresholds (57). AMBRA1 is at the intersection of autophagy and apoptosis; namely, AMBRA1 not only participates in autophagy but also plays roles in mitochondrial apoptosis (58). Along with the stress aggravation, AMBRA1-mediated autophagy fails to restore the normal function of the cell, and then the cell will initiate the apoptotic program. The full-length AMBRA1 is cleaved by caspases to remove the N terminus and turn into a pro-apoptotic BH3-like protein. The cleaved form of AMBRA1 binds and inhibits the activity of anti-apoptotic BCL2 family proteins BCL2, MCL1, and BCL2L1 to promote cell death (48). To sum up, AMBRA1 can simultaneously be a regulator in the process of autophagy and apoptosis.

Unfolded protein response (UPR) also regulates the cross-talk between autophagy and apoptosis (59). In general, activation of UPR promotes cell survival by inducing cytoprotective autophagy and inhibiting apoptosis (60). Cancer cells are often subjected to numerous intrinsic and extrinsic insults that result in the destruction of protein homeostasis (proteostasis) and the accumulation of unfolded or misfolded proteins in the endoplasmic reticulum (ER), which is known as ER stress (61). To counteract ER stress, cells activate a series of adaptive mechanisms of UPR to clear unfolded or misfolded proteins and restore proteostasis (62). UPR is controlled by three ER-transmembrane stress sensors, activating transcription factor 6 (ATF6), inositol-requiring enzyme 1α (IRE1α), and pancreatic endoplasmic reticulum kinase (PERK) (63). Under ER stress conditions, IRE1 phosphorylates BCL2 and BCL-XL by JUN N-terminal kinase (JNK), which promotes the dissociation of BECLIN1 and BCL2 families (59). Although the phosphorylation of BCL2 and BCL-XL by JNK has no effect on the binding between AMBRA1 and BCL2 proteins (18), UPR can also promote the activity of AMBRA1. Specifically, PERK inhibits mTORC1 by enhancing the expression of Tribbles Homolog 3 (TRB3) with the help of C/EBP homologous protein (CHOP) (59), while calcium release from ER also positively regulates the activity of AMPK (64), suggesting that UPR may activate the activity of AMBRA1 through both inhibiting mTORC1 and activating AMPK. However, if the insults are prolonged and severe, pro-survival UPR will transform into pro-apoptotic UPR. UPR can promote apoptosis by activating pro-apoptotic BCL2 proteins, BAX and BAK (65). The cleaved AMBRA1 can enhance the pro-apoptotic role of UPR by inhibiting the activity of anti-apoptotic BCL2 family proteins (48). These indicate that UPR and AMBRA1-mediated autophagy may coordinate with each other in modulating survival and apoptosis.

## Role of AMBRA1 in tumorigenesis and tumor progression

c-MYC belongs to the “super transcription factors” family and is deregulated in >50% of cancers, which is an important target for cancer therapy (66). AMBRA1 regulates the activity of c-MYC through different pathways, and the roles of AMBRA1 in regulating c-MYC are controversial. Cianfanelli et al. investigated the cross-talk between two mTOR-dependent cell processes, autophagy induction and proliferation suppression, through four different approaches: gene-trap mutation in *AMBRA1 locus*, siRNA interference, *Ambra1* heterozygous (*Ambra^+/gt^*) mice, and zebrafish embryo transplantation. The study finally identified that AMBRA1 interacts with the phosphatase PP2A and enhances its phosphatase activity on the proto-oncogene c-MYC, which further prevents tumorigenesis and tumor hyperproliferation (26). However, another study presented a different perspective that AMBRA1 is a tumor stemness-promoting factor in medulloblastoma (MB). Myc-Interacting Zinc Finger Protein 1 (MIZ-1) is a c-MYC cofactor, which is known to regulate AMBRA1 transcription directly (67). In MB subgroups of patients with enhanced levels of the c-MYC oncogene (MB_Group3_), c-MYC correlating with MIZ-1 promotes the transcription of AMBRA1. Consequently, AMBRA1 promotes the activity of c-MYC through SOCS3/STAT3 pathway, which contributes to MB_Group3_ stem potential, growth, and migration (23). The cancerous inhibitor of protein phosphatase 2A (CIP2A) is an oncoprotein that could inhibit PP2A and stabilize c-MYC in human malignancies (68). The mechanism of AMBRA1 and CIP2A in regulating c-MYC is similar in that they both are under the control of mTORC1 and regulate the activity of PP2A. AMBRA1 inhibits the activity of c-MYC by enhancing the activity of PP2A, which inhibits the proliferation and tumorigenesis of cancer, but CIP2A plays the opposite role on PP2A to AMBRA1 (69). However, whether there is a direct interaction between AMBRA1 and CIP2A is unknown.

In addition to c-MYC, the cell cycle protein cyclin D is another key target in cancer therapy (70). The cyclin D–cyclin-dependent kinase (CDK) 4/6 complex is the fundamental factor for cell cycle progression, which promotes the transition from the G0 or G1 to S phase temporally (71). Thus, the cyclin D–CDK4/6 complex is frequently overexpressed and hyperactivated in various cancers (72). Recently, there is a breakthrough in the mechanism of cyclin D decomposition. Three independent studies unveiled a novel ubiquitylation degradation mechanism of cyclin D. As a substrate receptor of the CULLIN 4/DDB1 complex, AMBRA1 directly binds and ubiquitinates cyclin D to promote its proteasomal degradation (34–36), while checkpoint kinase 1 (CHK1) is a key kinase in the replication stress response, and its inhibition aggravates DNA damage and leads to cell death in AMBRA1-null cancer cells (35). Furthermore, CDK2 is the catalytic subunit of the CDK complex, whose inhibition recovers the sensibility of AMBRA1-deficient tumors to CDK4/6 inhibitors palbociclib or abemaciclib (34). These findings elucidated therapeutic vulnerabilities in AMBRA1-deficient tumors and shed light on future clinical trials.

**Figure 4:**
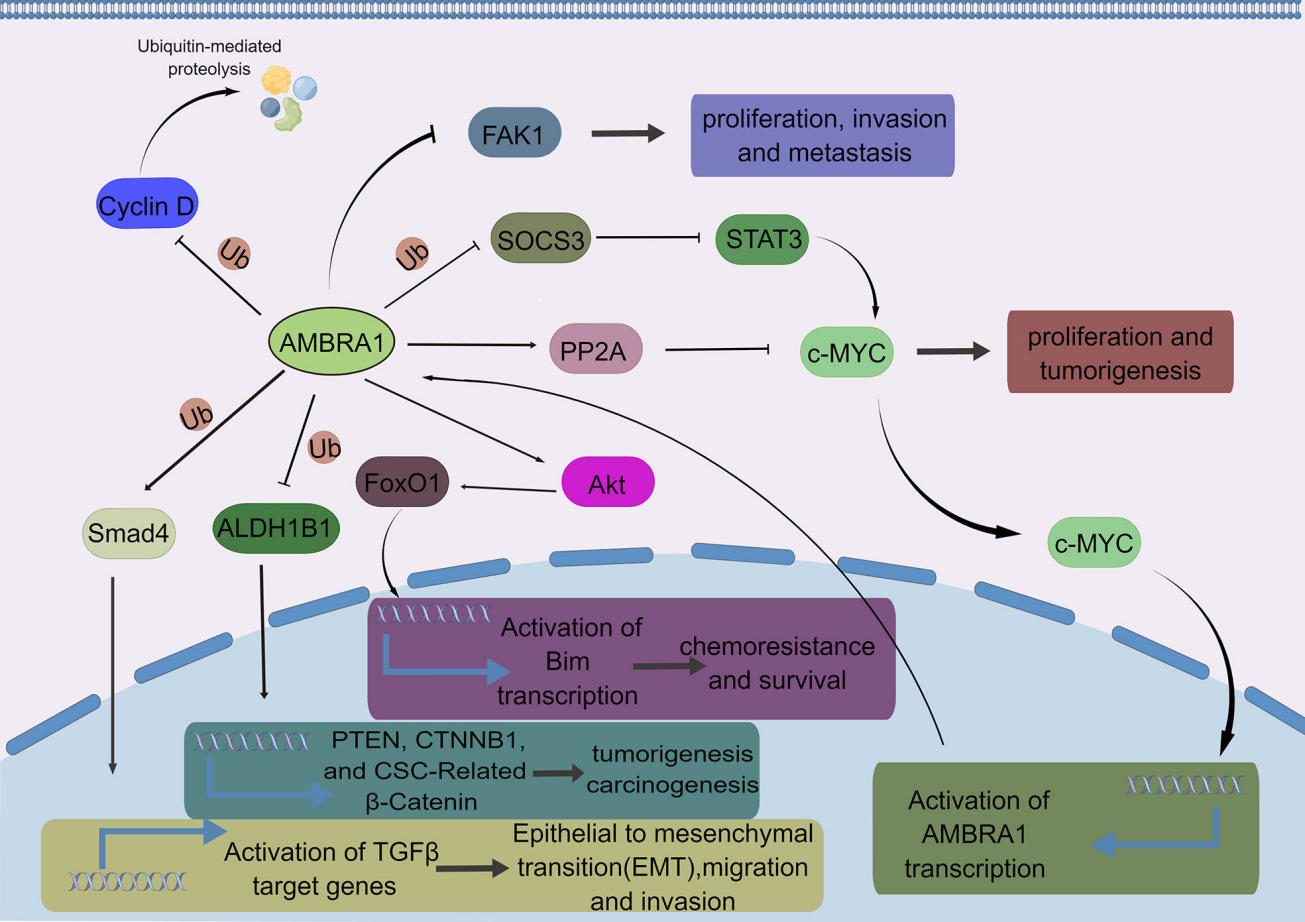
The major AMBRA1-related signaling pathways in cancer. AMBRA1 ubiquitinates cyclin D to promote its proteasomal degradation. AMBRA1 inhibits the proliferation, invasion, and metastasis of melanoma by inhibiting the phosphoactivation of FAK1. AMBRA1 inhibits the activity of c-MYC by enhancing the activity of PP2A, thereby inhibiting the proliferation and tumorigenesis of cancer cells, whereas AMBRA1 also promotes the activity of c-MYC through SOCS3/STAT3 pathway, enhancing tumor stem potential, growth, and migration of MBGroup3 stem cells. AMBRA1 promotes chemoresistance and survival in breast cancer cells through the AKT-FOXO1-BIM axis. AMBRA1 inhibits tumorigenesis and carcinogenesis by ubiquitylating ALDH1B1, a cancer stem cell marker. AMBRA1 mediates non-proteolytic polyubiquitylation of SMAD4 to enhance its transcriptional functions. Consequently, AMBRA1 potentiated TGFβ signaling and critically promoted TGFβ-induced epithelial-to-mesenchymal transition, migration, and invasion of breast cancer cells.

AMBRA1 is also associated with cancer development, including EMT, migration, invasion, and metastasis (26, 45, 73) (**Figure 4**). Interestingly, AMBRA1 plays an oncogenic role in hepatocellular carcinoma, metastatic breast cancer, and medulloblastoma, whereas AMBRA1 seems to be a tumor suppressor in colorectal cancer cell, melanoma, and squamous cell carcinoma (23, 24, 29, 33, 38, 45, 74, 75). The different effects of AMBRA1 in cancers may be due to diverse types and stages of cancer, as AMBRA1 is an autophagy-associated protein and has different roles depending on the gene context (76). Future studies need to focus on ascertaining the underlying mechanisms of how AMBRA1 plays opposite roles in different cancer types and figure out the gene context determining different functions of AMBRA1.

## AMBRA1—a potential target for anticancer therapy

One of the major barriers in anticancer therapies is attributed to tumor resistance to apoptosis (30). As mentioned above, AMBRA1, an autophagy-related protein, is the direct substrate of caspases and calpains and acts an important role in apoptosis as well (13, 48). However, AMBRA1 is at the crossroad between autophagy and apoptosis and might be a novel prognostic and therapeutic candidate target for cancer therapy.

The role of autophagy in cancer therapies remains controversial (76). AMBRA1 is an autophagy-related protein and plays an important role in autophagy induction, so it can enhance resistance or sensitivity to chemotherapeutic agents in cancer treatment. In general, AMBRA1-mediated autophagy is pro-tumoral. Specifically, AMBRA1-mediated autophagy reduces the sensitivity to cisplatin in pancreatic cancer cells, ovarian cancer cells, and oropharyngeal squamous cell carcinoma cells (77–79). Sun et al. reported that AMBRA1 inhibited paclitaxel-induced apoptosis and chemosensitivity *via* the AKT−FOXO1−BIM pathway in MCF-7 and MDA-MB-231 breast cancer cells (80, 81). The same group also found that AMBRA1 expression level was negatively correlated with the sensitivity of breast cancer cells to epirubicin previously (82). In contrast, AMBRA1-associated autophagy may also be anti-tumoral. Shen and colleagues unveiled that AMBRA1 was significantly upregulated in MDA-MB-231 and MDA-MB-453 cells after treating with cisplatin. While treating these cancer cells with a classic autophagy inhibitor 3-methyladenine (3-MA), they found that the cytotoxicity of cisplatin is impaired, which indicates that AMBRA1-mediated autophagy could enhance the cytotoxicity of cisplatin (83). Therefore, in the following studies, the identification of specific cell contexts in which AMBRA1-mediated autophagy exerts chemo-sensitization or resistance will be beneficial to potential AMBRA1 targeting therapies.

Radiation therapy is another classical cancer treatment scheme, and AMBRA1 also regulates tumor sensitivity to radiotherapy. AMBRA1-associated autophagy promotes the transition from hyper-radiosensitivity to induced radio-resistance in A549 and H460 human lung adenocarcinoma cell lines (84). Calcitriol, an active metabolite of vitamin D, enhances the sensitivity to irradiation in SiHa and CaSki cervical cancer cells by promoting AMBRA1 degradation (85). Therefore, the combination of AMBRA1 suppression and chemoradiotherapy may achieve a favorable outcome. Although the effect of AMBRA1-mediated autophagy on chemoradiotherapy is relatively limited at present, many studies indicated that BECLIN1 has a significant impact on chemoradiotherapy (86–89). Given that AMBRA1 is a vital component of the BECLIN1/VPS34 complex and regulates the activity of BECLIN1 (13), it is suggested that AMBRA1-mediated autophagy influences chemotherapy and radiotherapy. However, the difference between AMBRA1- and BECLIN1-associated autophagy needs to be further investigated.

In addition to the two classic treatments mentioned above, cancer immunotherapy as an emerging trajectory has played a more critical role in cancer therapy in the last decade. AMBRA1 is also involved in immune regulation, as AMBRA1 regulates the activities of various subtypes of T cells. Firstly, previous studies have shown that autophagy is associated with the survival, differentiation, and activation of T cells (90). Sato et al. found that AMBRA1 regulates the activity of OVA53 precursor T cells and naive T cells in an autophagy-dependent manner (91). Furthermore, this group also found that AMBRA1 regulates the proliferation of precursor T cells and naive T cells in an autophagy-independent manner (92). This regulation might be attributed to the recent finding that CULLIN4–AMBRA1 E3 ligase regulates the stability of cyclin D to control the cell cycle (35). Becher et al. also presented that AMBRA1 promotes differentiation and maintenance of human regulatory T cells by facilitating FOXP3 transcription *in vitro* and *in vivo* (27). The suppressor of cytokine signaling-3 (SOCS3) is a well-known feedback inhibitor of the JAK/STAT3 pathway, and STAT3 is central in regulating the anti-tumor immune response (93–95), while AMBRA1 activates STAT3 through suppression of SOCS3 in hepatocellular carcinoma and medulloblastoma (23, 24), indicating that the regulation of tumor immunogenicity by AMBRA1 may be in a STAT3-dependent manner.

Finally, a growing number of studies found that microRNAs (miRNAs), ~22-nt non-coding single-stranded RNAs, are directly associated with some important physiology and disease progression of plants and animals in a post-transcription modification manner. Insights into the roles of miRNAs in cancer have made miRNAs attractive targets for novel therapeutic approaches (96). By analyzing four independent databases (97–100) (DIANA-microT v5, TargetScan 8.0, microrna.org, and PicTar), only miR-23a-3p, miR-7-5p, miR-9-5p, and miR-200bc-3p/429 were identified as potential miRNAs targeting *AMBRA1*, suggesting the limited number of conserved miRNA binding sites in 3′-UTR of *AMBRA1* mRNA. These miRNAs can regulate chemosensitivity and cancer proliferation by targeting *AMBRA1* mRNA. MiR-23a-5p and miR-23a-3p derive from the same precursor miRNA-23a but are processed from the 5′ and 3′ arms, respectively. MiR-23a-5p restored the sensitivity of NB4 cells to arsenic trioxide (ATO) by targeting *AMBRA1*, and similar results were obtained in U937 cells. Moreover, clinical samples analysis revealed that miR-23a-5p is correlated with the NF-κB pathway in relapsed acute promyelocytic leukemia patients (101). MIR7–3HG promoted cell proliferation by targeting *AMBRA1* mRNA, which prevented c-MYC degradation to enhance transcription in HeLa cells and A549 cells (102). A recent study found that miRNA-198 targeted *AMBRA1* mRNA and regulated the enzalutamide-resistant prostate cancer growth *in vitro* and *in vivo* (103). Since miRNA is highly tissue-specific and can be used to predict molecular phenotypes of cancers, these specific miRNAs might be used as a basic approach to diagnose and treat cancers of AMBRA1 abnormity.

## Conclusions and perspectives

The incidence and mortality of cancer are increasing yearly, and cancer is the major source of the global disease burden. A systematic analysis estimates that the burden of cancer will continue to rise for at least the next 20 years (104). As the pathogenesis of tumors is complicated, tons of studies unveiled many mechanisms of tumor initiation and progression (105–109). AMBRA1 as an emerging haploinsufficient tumor suppressor plays a pivotal role in tumorigenesis and progression (26, 45). Furthermore, AMBRA1-mediated autophagy plays controversial roles in chemoradiotherapy (78, 79, 81–84), and the different roles of AMBRA1-associated autophagy in cancer treatment seem to depend on tumor type, stage, and genetic context (76).

AMBRA1 is an intrinsically disordered protein that was associated with various tumor progressions, including autophagy, tumorigenesis, proliferation, EMT, and apoptosis (26, 34, 45, 48). Studies found that AMBRA1 regulates tumorigenesis by targeting the activity of c-MYC, STAT3, and ALDH1B1 (23, 26, 33). In addition, AMBRA1 also regulated tumor proliferation, EMT, migration, and invasion by inhibiting cyclin D, FAK1, and Smad4 (34–36, 38, 45). However, these studies were mainly conducted *in vitro*; further work will be focused on validating these findings *in vivo*. In addition, it would be interesting to understand how AMBRA1 itself is regulated, both with relation to the cell cycle and in light of the multiple well-established functions. Moreover, several AMBRA1 isoforms are annotated in the human genome. It also remains to be determined whether these possible protein isoforms exist in cells and, if so, how their functions differ.

Currently, the post-translational modifications (PTMs) of AMBRA1 are only focused on phosphorylation and ubiquitylation (21, 22, 110), which are mainly associated with autophagy. To the best of our knowledge, there is almost no report about other types of PTMs, such as SUMOylation, methylation, and acetylation of AMBRA1, to date. Potential PTM forms of AMBRA1 may be identified through mass spectrometry and investigated in various physiological and pathological conditions.

In sum, targeting AMBRA1 has the potential to inhibit tumorigenesis and tumor progression in some types of malignancies. Furthermore, AMBRA1 tightly correlates with chemoresistance. During chemotherapy, cancer cells could attenuate the cytotoxicity of chemotherapeutic agents through autophagy, thereby promoting cancer survival. Therefore, autophagy inhibition by targeting AMBRA1 might enhance the effect of agents to achieve the therapeutic goal.

## Author contributions

XL wrote the manuscript. XW read and approved the final manuscript. YL and JL revised the manuscript, and provided suggestions for the final manuscript. All authors read and approved the manuscript and agreed to be accountable for all aspects of the research in ensuring that the accuracy or integrity of any part of the work were appropriately investigated and resolved.

## Funding

This work is supported by the National Natural Science Foundation of China (81972361) and Natural Science Foundation of Henan Province (182300410379).

## Conflict of interest

The authors declare that the research was conducted in the absence of any commercial or financial relationships that could be construed as a potential conflict of interest.

## Publisher’s note

All claims expressed in this article are solely those of the authors and do not necessarily represent those of their affiliated organizations, or those of the publisher, the editors and the reviewers. Any product that may be evaluated in this article, or claim that may be made by its manufacturer, is not guaranteed or endorsed by the publisher.

## References

[1] Sung H Ferlay J Siegel RL Laversanne M Soerjomataram I Jemal A. Global cancer statistics 2020: GLOBOCAN estimates of incidence and mortality worldwide for 36 cancers in 185 countries. CA Cancer J Clin (2021) 71(3):209–49. doi: 10.3322/caac.21660 33538338

[2] Bray F Laversanne M Weiderpass E Soerjomataram I. The ever-increasing importance of cancer as a leading cause of premature death worldwide. Cancer (2021) 127(16):3029–30. doi: 10.1002/cncr.33587 34086348

[3] Siegel RL Miller KD Fuchs HE Jemal A. Cancer statistics, 2022. CA Cancer J Clin (2022) 72(1):7–33. doi: 10.3322/caac.21708 35020204

[4] Son B Lee S Youn H Kim E Kim W Youn B. The role of tumor microenvironment in therapeutic resistance. Oncotarget (2017) 8(3):3933–45. doi: 10.18632/oncotarget.13907 PMC5354804 27965469

[5] Ippolito MR Martis V Martin S Tijhuis AE Hong C Wardenaar R. Gene copy-number changes and chromosomal instability induced by aneuploidy confer resistance to chemotherapy. Dev Cell (2021) 56(17):2440–2454.e6. doi: 10.1016/j.devcel.2021.07.006 34352223

[6] Gradishar WJ Anderson BO Abraham J Aft R Agnese D Allison KH. Breast cancer, version 3.2020, NCCN clinical practice guidelines in oncology. J Natl Compr Canc Netw (2020) 18(4):452–78. doi: 10.6004/jnccn20200016 32259783

[7] Baehrecke EH. Autophagy: dual roles in life and death? Nat Rev Mol Cell Biol (2005) 6(6):505–10. doi: 10.1038/nrm1666 15928714

[8] Kroemer G Levine B. Autophagic cell death: The story of a misnomer. Nat Rev Mol Cell Biol (2008) 9(12):1004–10. doi: 10.1038/nrm2529 PMC2727358 18971948

[9] Ashrafi G Schwarz TL. The pathways of mitophagy for quality control and clearance of mitochondria. Cell Death Differ (2013) 20(1):31–42. doi: 10.1038/cdd.2012.81 22743996 PMC3524633

[10] Kunz JB Schwarz H Mayer A. Determination of four sequential stages during microautophagy *in vitro* . J Biol Chem (2004) 279(11):9987–96. doi: 10.1074/jbc.M307905200 14679207

[11] Majeski AE Dice JF. Mechanisms of chaperone-mediated autophagy. Int J Biochem Cell Biol (2004) 36(12):2435–44. doi: 10.1016/j.biocel.2004.02.013 15325583

[12] Li X He S Ma B. Autophagy and autophagy-related proteins in cancer. Mol Cancer (2020) 19(1):12. doi: 10.1186/s12943-020-1138-4 31969156 PMC6975070

[13] Fimia GM Stoykova A Romagnoli A Giunta L Di Bartolomeo S Nardacci R. Ambra1 regulates autophagy and development of the nervous system. Nature (2007) 447(7148):1121–5. doi: 10.1038/nature05925 17589504

[14] Mei Y Su M Soni G Salem S Colbert CL Sinha SC. Intrinsically disordered regions in autophagy proteins. Proteins (2014) 82(4):565–78. doi: 10.1002/prot.24424 PMC3949125 24115198

[15] Dyson HJ Wright PE. Intrinsically unstructured proteins and their functions. Nat Rev Mol Cell Biol (2005) 6(3):197–208. doi: 10.1038/nrm1589 15738986

[16] Cianfanelli V De Zio D Di Bartolomeo S Nazio F Strappazzon F Cecconi F. Ambra1 at a glance. J Cell Sci (2015) 128(11):2003–8. doi: 10.1242/jcs.168153 26034061

[17] Di Bartolomeo S Corazzari M Nazio F Oliverio S Lisi G Antonioli M. The dynamic interaction of AMBRA1 with the dynein motor complex regulates mammalian autophagy. J Cell Biol (2010) 191(1):155–68. doi: 10.1083/jcb.201002100 PMC2953445 20921139

[18] Strappazzon F Vietri-Rudan M Campello S Nazio F Florenzano F Fimia GM. Mitochondrial BCL-2 inhibits AMBRA1-induced autophagy. EMBO J (2011) 30(7):1195–208. doi: 10.1038/emboj.2011.49 PMC3094111 21358617

[19] Van Humbeeck C Cornelissen T Hofkens H Mandemakers W Gevaert K De Strooper B. Parkin interacts with Ambra1 to induce mitophagy. J Neurosci (2011) 31(28):10249–61. doi: 10.1523/JNEUROSCI.1917-11.2011 PMC6623066 21753002

[20] Pagliarini V Wirawan E Romagnoli A Ciccosanti F Lisi G Lippens S. Proteolysis of Ambra1 during apoptosis has a role in the inhibition of the autophagic pro-survival response. Cell Death Differ (2012) 19(9):1495–504. doi: 10.1038/cdd.2012.27 PMC3422474 22441670

[21] Nazio F Strappazzon F Antonioli M Bielli P Cianfanelli V Bordi M. mTOR inhibits autophagy by controlling ULK1 ubiquitylation, self-association and function through AMBRA1 and TRAF6. Nat Cell Biol (2013) 15(4):406–16. doi: 10.1038/ncb2708 23524951

[22] Antonioli M Albiero F Nazio F Vescovo T Perdomo AB Corazzari M. AMBRA1 interplay with cullin E3 ubiquitin ligases regulates autophagy dynamics. Dev Cell (2014) 31(6):734–46. doi: 10.1016/j.devcel.2014.11.013 25499913

[23] Nazio F Po A Abballe L Ballabio C Camassei FD Bordi M. Targeting cancer stem cells in medulloblastoma by inhibiting AMBRA1 dual function in autophagy and STAT3 signalling. Acta Neuropathologica (2021) 142(3):537–64. doi: 10.1007/s00401-021-02347-7 PMC8357694 34302498

[24] Chen S-H Jang GM Huttenhain R Gordon DE Du D Newton BW. CRL4(AMBRA1) targets elongin c for ubiquitination and degradation to modulate CRL5 signaling. EMBO J (2018) 37(18):e97508. doi: 10.15252/embj.201797508 30166453 PMC6138441

[25] Xia P Wang S Huang G Du Y Zhu P Li M. RNF2 is recruited by WASH to ubiquitinate AMBRA1 leading to downregulation of autophagy. Cell Res (2014) 24(8):943–58. doi: 10.1038/cr.2014.85 PMC4123297 24980959

[26] Cianfanelli V Fuoco C Lorente M Salazar M Quondamatteo F Gherardini PF. AMBRA1 links autophagy to cell proliferation and tumorigenesis by promoting c-myc dephosphorylation and degradation. Nat Cell Biol (2015) 17(1):20. doi: 10.1038/ncb3072 25438055 PMC4976803

[27] Becher J Simula L Volpe E Procaccini C La Rocca C D'Acunzo P. AMBRA1 controls regulatory T-cell differentiation and homeostasis upstream of the FOXO3-FOXP3 axis. Dev Cell (2018) 47(5):592–607.e6. doi: 10.1016/j.devcel.2018.11.010 30513302

[28] Strappazzon F Nazio F Corrado M Cianfanelli V Romagnoli A Fimia GM. AMBRA1 is able to induce mitophagy via LC3 binding, regardless of PARKIN and p62/SQSTM1. Cell Death Differ (2015) 22(3):419. doi: 10.1038/cdd2014139 25215947 PMC4326570

[29] Schoenherr C Byron A Griffith B Loftus A Wills JC Munro AF. The autophagy protein Ambra1 regulates gene expression by supporting novel transcriptional complexes. J Biol Chem (2020) 295(34):12045–57. doi: 10.1074/jbc.RA120.012565 PMC7443501 32616651

[30] Mohammad RM Muqbil I Lowe L Yedjou C Hsu HY Lin LT. Broad targeting of resistance to apoptosis in cancer. Semin Cancer Biol (2015) 35 Suppl(0):S78–S103. doi: 10.1016/j.semcancer.2015.03.001 25936818 PMC4720504

[31] Di Rita A Peschiaroli A D'Acunzo P Strobbe D Hu Z Gruber J. HUWE1 E3 ligase promotes PINK1/PARKIN-independent mitophagy by regulating AMBRA1 activation via IKK alpha. Nat Commun (2018) 9:3755. doi: 10.1038/s41467-018-05722-3 30217973 PMC6138665

[32] Strappazzon F Rita Di A Peschiaroli A Leoncini PP Locatelli F Melino G. HUWE1 controls MCL1 stability to unleash AMBRA1-induced mitophagy. Cell Death Differ (2020) 27(4):1155. doi: 10.1038/s41418-019-0404-8 31434979 PMC7206129

[33] Baek S-H Jang Y-K. AMBRA1 negatively regulates the function of ALDH1B1, a cancer stem cell marker, by controlling its ubiquitination. Int J Mol Sci (2021) 22(21):12079. doi: 10.3390/ijms222112079 34769507 PMC8584921

[34] Simoneschi D Rona G Zhou N Jeong YT Jiang S Milletti G. CRL4(AMBRA1) is a master regulator of d-type cyclins. Nature (2021) 592(7856):789–93. doi: 10.1038/s41586-021-03445-y PMC8875297 33854235

[35] Maiani E Milletti G Nazio F Holdgaard SG Bartkova J Rizza S. AMBRA1 regulates cyclin d to guard s-phase entry and genomic integrity. Nature (2021) 592(7856):799–803. doi: 10.1038/s41586-021-03422-5 33854232 PMC8864551

[36] Chaikovsky AC Li C Jeng EE Loebell S Lee MC Murray CW. The AMBRA1 E3 ligase adaptor regulates the stability of cyclin d. Nature (2021) 592(7856):794–8. doi: 10.1038/s41586-021-03474-7 PMC8246597 33854239

[37] Di Rienzo M Romagnoli A Ciccosanti F Refolo G Consalvi V Arena G. AMBRA1 regulates mitophagy by interacting with ATAD3A and promoting PINK1 stability. Autophagy (2022) 18(8):1752–62. doi: 10.1080/1554862720211997052 PMC9450973 34798798

[38] Liu J Yuan B Cao J Luo H Gu S Zhang M. AMBRA1 promotes TGFβ signaling via nonproteolytic polyubiquitylation of Smad4. Cancer Res (2021) 81(19):5007–20. doi: 10.1158/0008-5472.CAN-21-0431 34362797

[39] Manganelli V Capozzi A Recalchi S Riitano G Mattei V Longo A. The role of cardiolipin as a scaffold mitochondrial phospholipid in autophagosome formation: *In vitro* evidence. Biomolecules (2021) 11(2):222. doi: 10.3390/biom11020222 33562550 PMC7915802

[40] Manganelli V Matarrese P Antonioli M Gambardella L Vescovo T Gretzmeier C. Raft-like lipid microdomains drive autophagy initiation via AMBRA1-ERLIN1 molecular association within MAMs. Autophagy (2021) 17(9):2528–48. doi: 10.1080/15548627.2020.1834207 PMC8496542 33034545

[41] Garofalo T Matarrese P Manganelli V Marconi M Tinari A Gambardella L. Evidence for the involvement of lipid rafts localized at the ER-mitochondria associated membranes in autophagosome formation. Autophagy (2016) 12(6):917–35. doi: 10.1080/15548627.2016.1160971 PMC4922444 27123544

[42] Cianfanelli V D’Orazio M Cecconi F. AMBRA1 and BECLIN 1 interplay in the crosstalk between autophagy and cell proliferation. Cell Cycle (2015) 14(7):959–63. doi: 10.1080/15384101.2015.1021526 PMC4615147 25803737

[43] Martorana F Gaglio D Bianco MR Aprea F Virtuoso A Bonanomi M. Differentiation by nerve growth factor (NGF) involves mechanisms of crosstalk between energy homeostasis and mitochondrial remodeling. Cell Death Dis (2018) 9:391. doi: 10.1038/s41419-018-0429-9 29523844 PMC5844953

[44] Ellis R Tang D Nasr B Greenwood A McConnell A Anagnostou ME. Epidermal autophagy and beclin 1 regulator 1 and loricrin: A paradigm shift in the prognostication and stratification of the American joint committee on cancer stage I melanomas. Br J Dermatol (2020) 182(1):156–65. doi: 10.1111/bjd18086 PMC6973157 31056744

[45] Di Leo L Bodemeyer V Bosisio FM Claps G Carretta M Rizza S. Loss of Ambra1 promotes melanoma growth and invasion. Nat Commun (2021) 12(1):2550. doi: 10.1038/s41467-021-22772-2 33953176 PMC8100102

[46] Cecconi F Bartolomeo Di S Nardacci R Fuoco C Corazzari M Giunta L. A novel role for autophagy in neurodevelopment. Autophagy (2007) 3(5):506–8. doi: 10.4161/auto.4616 17622796

[47] Jain BP Pandey S. WD40 repeat proteins: Signalling scaffold with diverse functions. Protein J (2018) 37(5):391–406. doi: 10.1007/s10930-018-9785-7 30069656

[48] Strappazzon F Rita Di A Cianfanelli V D'Orazio M Nazio F Fimia GM. Prosurvival AMBRA1 turns into a proapoptotic BH3-like protein during mitochondrial apoptosis. Autophagy (2016) 12(6):963–75. doi: 10.1080/15548627.2016.1164359 PMC4922440 27123694

[49] Di Rita A Strappazzon F. AMBRA1, a novel BH3-like protein: New insights into the AMBRA1-BCL2-Family proteins relationship. Int Rev Cell Mol Biol (2017) 330:85–113. doi: 10.1016/bs.ircmb.2016.09.002 28215535

[50] Clark SL Jr. Cellular differentiation in the kidneys of newborn mice studies with the electron microscope. J Biophys Biochem Cytol (1957) 3(3):349–62. doi: 10.1083/jcb.3.3.349 PMC2224034 13438920

[51] Deter RL Baudhuin P De Duve C. Participation of lysosomes in cellular autophagy induced in rat liver by glucagon. J Cell Biol (1967) 35(2):C11–6. doi: 10.1083/jcb.35.2.C11 PMC2107130 6055998

[52] Ohsumi Y. Molecular dissection of autophagy: Two ubiquitin-like systems. Nat Rev Mol Cell Biol (2001) 2(3):211–6. doi: 10.1038/35056522 11265251

[53] Suzuki K Ohsumi Y. Molecular machinery of autophagosome formation in yeast, saccharomyces cerevisiae. FEBS Lett (2007) 581(11):2156–61. doi: 10.1016/j.febslet.2007.01.096 17382324

[54] Kim J Kundu M Viollet B Guan KL. AMPK and mTOR regulate autophagy through direct phosphorylation of Ulk1. Nat Cell Biol (2011) 13(2):132–41. doi: 10.1038/ncb2152 PMC3987946 21258367

[55] Axe EL Walker SA Manifava M Chandra P Roderick HL Habermann A. Autophagosome formation from membrane compartments enriched in phosphatidylinositol 3-phosphate and dynamically connected to the endoplasmic reticulum. J Cell Biol (2008) 182(4):685–701. doi: 10.1083/jcb.200803137 18725538 PMC2518708

[56] Onishi M Yamano K Sato M Matsuda N Okamoto K. Molecular mechanisms and physiological functions of mitophagy. EMBO J (2021) 40(3):e104705. doi: 10.15252/embj.2020104705 33438778 PMC7849173

[57] Maiuri MC Zalckvar E Kimchi A Kroemer G. Self-eating and self-killing: crosstalk between autophagy and apoptosis. Nat Rev Mol Cell Biol (2007) 8(9):741–52. doi: 10.1038/nrm2239 17717517

[58] Fimia GM Corazzari M Antonioli M Piacentini M. Ambra1 at the crossroad between autophagy and cell death. Oncogene (2013) 32(28):3311–8. doi: 10.1038/onc.2012.455 23069654

[59] Clarke R Cook KL Hu R Facey CO Tavassoly I Schwartz JL. Endoplasmic reticulum stress, the unfolded protein response, autophagy, and the integrated regulation of breast cancer cell fate. Cancer Res (2012) 72(6):1321–31. doi: 10.1158/0008-5472.CAN-11-3213 PMC3313080 22422988

[60] Bhardwaj M Leli NM Koumenis C Amaravadi RK. Regulation of autophagy by canonical and non-canonical ER stress responses. Semin Cancer Biol (2020) 66:116–28. doi: 10.1016/j.semcancer.2019.11.007 PMC7325862 31838023

[61] Urra H Dufey E Avril T Chevet E Hetz C. Endoplasmic reticulum stress and the hallmarks of cancer. Trends Cancer (2016) 2(5):252–62. doi: 10.1016/j.trecan.2016.03.007 28741511

[62] Hetz C. The unfolded protein response: controlling cell fate decisions under ER stress and beyond. Nat Rev Mol Cell Biol (2012) 13(2):89–102. doi: 10.1038/nrm3270 22251901

[63] Hetz C Zhang K Kaufman RJ. Mechanisms, regulation and functions of the unfolded protein response. Nat Rev Mol Cell Biol (2020) 21(8):421–38. doi: 10.1038/s41580-020-0250-z PMC8867924 32457508

[64] Verfaillie T Salazar M Velasco G Agostinis P. Linking ER stress to autophagy: Potential implications for cancer therapy. Int J Cell Biol (2010) 2010(1):930509. doi: 10.1155/2010/930509 20145727 PMC2817393

[65] Hetz C Bernasconi P Fisher J Lee AH Bassik MC Antonsson B. Proapoptotic BAX and BAK modulate the unfolded protein response by a direct interaction with IRE1alpha. Science (2006) 312(5773):572–6. doi: 10.1126/science.1123480 16645094

[66] Duffy MJ O'Grady S Tang M Crown J. MYC as a target for cancer treatment. Cancer Treat Rev (2021) 94:102154. doi: 10.1016/j.ctrv.2021.102154 33524794

[67] Wolf E Gebhardt A Kawauchi D Walz S Eyss von B Wagner N. Miz1 is required to maintain autophagic flux. Nat Commun (2013) 4:2535. doi: 10.1038/ncomms3535 24088869 PMC4084558

[68] Junttila MR Puustinen P Niemelä M Ahola R Arnold H Böttzauw T. CIP2A inhibits PP2A in human malignancies. Cell (2007) 130(1):51–62. doi: 10.1016/j.cell.2007.04.044 17632056

[69] Puustinen P Rytter A Mortensen M Kohonen P Moreira JM Jäättelä M. CIP2A oncoprotein controls cell growth and autophagy through mTORC1 activation. J Cell Biol (2014) 204(5):713–27. doi: 10.1083/jcb.201304012 PMC3941044 24590173

[70] Otto T Sicinski P. Cell cycle proteins as promising targets in cancer therapy. Nat Rev Cancer (2017) 17(2):93–115. doi: 10.1038/nrc.2016.138 28127048 PMC5345933

[71] Witkiewicz AK Kumarasamy V Sanidas I Knudsen ES. Cancer cell cycle dystopia: Heterogeneity, plasticity, and therapy. Trends Cancer (2022) 8(9):711–725. doi: 10.1016/jtrecan202204006 PMC9388619 35599231

[72] Suski JM Braun M Strmiska V Sicinski P. Targeting cell-cycle machinery in cancer. Cancer Cell (2021) 39(6):759–78. doi: 10.1016/jccell202103010 PMC8206013 33891890

[73] Tang DYL Ellis RA Lovat PE. Prognostic impact of autophagy biomarkers for cutaneous melanoma. Front Oncol (2016) 6(9):236. doi: 10.3389/fonc201600236. 27882308 PMC5101199

[74] Schoenherr C Byron A Sandilands E Paliashvili K Baillie GS Valacca C. Ambra1 spatially regulates src activity and Src/FAK-mediated cancer cell invasion via trafficking networks. Elife (2017) 6:e23172. doi: 10.7554/eLife23172 28362576 PMC5376188

[75] Cosgarea I McConnell AT Ewen T Tang D Hill DS Anagnostou M. Melanoma secretion of TGFbeta-2 leads to loss of epidermal AMBRA1 threatening epidermal integrity and facilitating tumour ulceration. Br J Dermatol (2021) 186(4):694–704. doi: 10.1111/bjd20889 PMC9546516 34773645

[76] White E DiPaola RS. The double-edged sword of autophagy modulation in cancer. Clin Cancer Res (2009) 15(17):5308–16. doi: 10.1158/1078-0432.CCR-07-5023 PMC2737083 19706824

[77] Li X Zhang L Yu L Wei W Lin X Hou X. shRNA-mediated AMBRA1 knockdown reduces the cisplatin-induced autophagy and sensitizes ovarian cancer cells to cisplatin. J Toxicol Sci (2016) 41(1):45–53. doi: 10.2131/jts.41.45 26763392

[78] Liu J Chen Z Guo J Wang L Liu X. Ambra1 induces autophagy and desensitizes human prostate cancer cells to cisplatin. Bioscience Rep (2019) 39:BSR20170770. doi: 10.1042/BSR20170770 PMC6706594 29101240

[79] Antonioli M Pagni B Vescovo T Ellis R Cosway B Rollo F. HPV sensitizes OPSCC cells to cisplatin-induced apoptosis by inhibiting autophagy through E7-mediated degradation of AMBRA1. Autophagy (2021) 17(10):2842–55. doi: 10.1080/15548627.2020.1847444 PMC8526016 33172332

[80] Sun WL Wang L Luo J Zhu HW Cai ZW. Ambra1 inhibits paclitaxel-induced apoptosis in breast cancer cells by modulating the bim/mitochondrial pathway. Neoplasma (2019) 66(3):377–85. doi: 10.4149/neo_2018_180710N467 30784282

[81] Sun WL He LY Liang L Liu SY Luo J Lv ML. Ambra1 regulates apoptosis and chemosensitivity in breast cancer cells through the akt-FoxO1-Bim pathway. Apoptosis (2022) 27(5-6):329–41. doi: 10.1007/s10495-022-01718-z 35257265

[82] Sun W-L Wang L Luo J Zhu H-W Cai Z-W. Ambra1 modulates the sensitivity of breast cancer cells to epirubicin by regulating autophagy via ATG12. Cancer Sci (2018) 109(10):3129–38. doi: 10.1111/cas13743 PMC6172055 30027574

[83] Shen M Duan W-M Wu M-Y Wang W-J Liu L Xu M-D. Participation of autophagy in the cytotoxicity against breast cancer cells by cisplatin. Oncol Rep (2015) 34(1):359–67. doi: 10.3892/or20154005 26005215

[84] Wang Q Chen Y Chang H Hu T Wang J Xie Y. The role and mechanism of ATM-mediated autophagy in the transition from hyper-radiosensitivity to induced radioresistance in lung cancer under low-dose radiation. Front Cell Dev Biol (2021) 9(5):650819. doi: 10.3389/fcell2021650819 34055781 PMC8149741

[85] Cheng G Liu B Yu X. Calcitriol sensitizes cervical cancer cells to irradiation by regulating autophagy and apoptosis. Int J Radiat OncologyBiologyPhysics (2020) 108(3, Supplement):e508. doi: 101016/jijrobp2020071601

[86] Huang T Wan X Alvarez AA James CD Song X Yang Y. MIR93 (microRNA -93) regulates tumorigenicity and therapy response of glioblastoma by targeting autophagy. Autophagy (2019) 15(6):1100–11. doi: 10.1080/15548627.2019.1569947 PMC6526840 30654687

[87] Chen W Li Z Liu H Jiang S Wang G Sun L. MicroRNA-30a targets BECLIN-1 to inactivate autophagy and sensitizes gastrointestinal stromal tumor cells to imatinib. Cell Death Dis (2020) 11(3):198. doi: 10.1038/s41419-020-2390-7 32251287 PMC7090062

[88] Salwa A Ferraresi A Chinthakindi M Vallino L Vidoni C Dhanasekaran DN. BECN1 and BRCA1 deficiency sensitizes ovarian cancer to platinum therapy and confers better prognosis. Biomedicines (2021) 9(2):207. doi: 10.3390/biomedicines9020207 33670664 PMC7922320

[89] Hu F Song D Yan Y Huang C Shen C Lan J. IL-6 regulates autophagy and chemotherapy resistance by promoting BECN1 phosphorylation. Nat Commun (2021) 12(1):3651. doi: 10.1038/s41467-021-23923-1 34131122 PMC8206314

[90] Clarke AJ Simon AK. Autophagy in the renewal, differentiation and homeostasis of immune cells. Nat Rev Immunol (2019) 19(3):170–83. doi: 10.1038/s41577-018-0095-2 30531943

[91] Akatsuka H Kuga S Masuhara K Davaadorj O Okada C Iida Y. AMBRAI is involved in T cell receptor-mediated metabolic reprogramming through an ATG7-independent pathway. Biochem Biophys Res Commun (2017) 491(4):1098–104. doi: 10.1016/j.bbrc.2017.08.019 28789945

[92] Masuhara K Akatsuka H Tokusanai M Li C Iida Y Okada Y. AMBRA1 controls antigen-driven activation and proliferation of naive T cells. Int Immunol (2021) 33(2):107–18. doi: 10.1093/intimm/dxaa063 32909612

[93] Kortylewski M Kujawski M Wang T Wei S Zhang S Pilon-Thomas S. Inhibiting Stat3 signaling in the hematopoietic system elicits multicomponent antitumor immunity. Nat Med (2005) 11(12):1314–21. doi: 10.1038/nm1325 16288283

[94] Villarino AV Kanno Y O’Shea JJ. Mechanisms and consequences of jak-STAT signaling in the immune system. Nat Immunol (2017) 18(4):374–84. doi: 10.1038/ni.3691 PMC11565648 28323260

[95] Zou S Tong Q Liu B Huang W Tian Y Fu X. Targeting STAT3 in cancer immunotherapy. Mol Cancer (2020) 19(1):145. doi: 10.1186/s12943-020-01258-7 32972405 PMC7513516

[96] Rupaimoole R Slack FJ. MicroRNA therapeutics: Towards a new era for the management of cancer and other diseases. Nat Rev Drug Discovery (2017) 16(3):203–22. doi: 10.1038/nrd.2016.246 28209991

[97] Maragkakis M Reczko M Simossis VA Alexiou P Papadopoulos GL Dalamagas T. DIANA-microT web server: elucidating microRNA functions through target prediction. Nucleic Acids Res (2009) 37(Web Server issue):W273–6. doi: 10.1093/nar/gkp292 PMC2703977 19406924

[98] Grimson A Farh KK Johnston WK Garrett-Engele P Lim LP Bartel DP. MicroRNA targeting specificity in mammals: Determinants beyond seed pairing. Mol Cell (2007) 27(1):91–105. doi: 10.1016/j.molcel.2007.06.017 17612493 PMC3800283

[99] Friedman RC Farh KK Burge CB Bartel DP. Most mammalian mRNAs are conserved targets of microRNAs. Genome Res (2009) 19(1):92–105. doi: 10.1101/gr.082701.108 18955434 PMC2612969

[100] Betel D Wilson M Gabow A Marks DS Sander C. The microRNA.org resource: targets and expression. Nucleic Acids Res (2008) 36(Database issue):D149–53. doi: 10.1093/nar/gkm995 PMC2238905 18158296

[101] Ganesan S Palani HK Lakshmanan V Balasundaram N Alex AA David S. Stromal-cells downregulate MiR-23a-5p levels in myeloid leukemic cells to activate protective-autophagy against chemotherapeutic agents. Blood (2017) 130(Supplement 1):3780–0. doi: 10.1038/s41419-019-1964-8

[102] Capizzi M Strappazzon F Cianfanelli V Papaleo E Cecconi F. MIR7-3HG, a MYC-dependent modulator of cell proliferation, inhibits autophagy by a regulatory loop involving AMBRA1. Autophagy (2017) 13(3):554–66. doi: 10.1080/15548627.2016.1269989 PMC5361610 28059583

[103] Chen L Sun Y Tang M Wu D Xiang Z Huang CP. High-dose-androgen-induced autophagic cell death to suppress the enzalutamide-resistant prostate cancer growth via altering the circRNA-BCL2/miRNA-198/AMBRA1 signaling. Cell Death Discovery (2022) 8(1):128. doi: 10.1038/s41420-022-00898-6 35318303 PMC8941094

[104] Kocarnik JM Compton K Dean FE Fu W Gaw BL Harvey JD. Cancer incidence, mortality, years of life lost, years lived with disability, and disability-adjusted life years for 29 cancer groups from 2010 to 2019: A systematic analysis for the global burden of disease study 2019. JAMA Oncol (2022) 8(3):420–44. doi: 10.1001/jamaoncol20216987 PMC8719276 34967848

[105] Qian BZ Pollard JW. Macrophage diversity enhances tumor progression and metastasis. Cell (2010) 141(1):39–51. doi: 10.1016/j.cell.2010.03.014 20371344 PMC4994190

[106] De Craene B Berx G. Regulatory networks defining EMT during cancer initiation and progression. Nat Rev Cancer (2013) 13(2):97–110. doi: 10.1038/nrc3447 23344542

[107] Mashouri L Yousefi H Aref AR Ahadi AM Molaei F Alahari SK. Exosomes: composition, biogenesis, and mechanisms in cancer metastasis and drug resistance. Mol Cancer (2019) 18(1):75. doi: 10.1186/s12943-019-0991-5 30940145 PMC6444571

[108] Gupta PB Pastushenko I Skibinski A Blanpain C Kuperwasser C. Phenotypic plasticity: Driver of cancer initiation, progression, and therapy resistance. Cell Stem Cell (2019) 24(1):65–78. doi: 10.1016/j.stem.2018.11.011 30554963 PMC7297507

[109] Huang T Song X Xu D Tiek D Goenka A Wu B. Stem cell programs in cancer initiation, progression, and therapy resistance. Theranostics (2020) 10(19):8721–43. doi: 10.7150/thno.41648 PMC7392012 32754274

[110] Cianfanelli V Nazio F Cecconi F. Connecting autophagy: AMBRA1 and its network of regulation. Mol Cell Oncol (2015) 2(1):e970059. doi: 10.4161/23723548.2014.970059 27308402 PMC4905234

